# Evaluating Interactive Language for Children with Autism Spectrum Disorder (ASD) in Different Contexts

**DOI:** 10.3390/children9060787

**Published:** 2022-05-27

**Authors:** Jinhuan Yang, Wentao Gu, Chen Feng

**Affiliations:** 1 School of Chinese Language and Literature, Nanjing Normal University, Nanjing 210097, China; yjhyangjinhuan@163.com; 2 School of Communication Sciences and Disorders, McGill University, Montreal, QC H3A 1G1, Canada; 3 Department of Humanities & Tourism, Rizhao Polytechnic, Rizhao 276800, China; fengchenbh@163.com

**Keywords:** autism spectrum disorder (ASD), structural language, pragmatic skills, contexts

## Abstract

Autism spectrum disorder (ASD) is characterized by impairments in the use of appropriate interactive language (including structural language and pragmatic skills) in social contexts. However, the phenotype and causes of interactive language deficits in children with ASD, in different contexts, are still unclear. In this study, we examined the structural language and pragmatic skills of children with ASD in four contexts: playing, drawing, reading, and free talking. We found that while children with ASD did not exhibit deficits in structural language (e.g., vocabulary and utterance), they clearly exhibit deficits in pragmatic skills. We, also, found that contexts played a key role in the use of interactive language by children with ASD. For example, the reading context had a significant impact on the diversity of vocabulary, while the playing and drawing contexts made an important contribution to the formation of complex utterances. The free talking context, on the other hand, contributed to producing more turns. Furthermore, Spearman’s rank correlation analysis was used to examine the relationships between maternal input and children’s language output. We found that the correlations between structural language and maternal input in children with ASD were not as high as revealed in previous studies, while a, relatively, obvious relationship was found between pragmatic skills and maternal input. Specifically, the total number of turns (TNT) for a child with ASD is related to their mother’s TNT, as are the total number of words (TNW) and number of different words (NDW). These results suggest that (1) assessment of pragmatic skills should be included in the evaluation of children with suspected ASD (2) the influence of context on pragmatic skills needs to be taken into account, when assessing the pragmatic development of children with ASD; and (3) the impact of maternal language on children’s language use is of great importance, for children with ASD.

## 1. Introduction

Autism spectrum disorder (ASD) is characterized by impairments in social communication and interaction as well as atypical restricted and repetitive patterns of behavior [1]. Social communication includes nonverbal communication and verbal interaction, while the deficit in verbal interaction in children with ASD manifests mainly as pragmatic impairments. The primary trigger for parents of potential children with ASD to initially seek a diagnosis is, often, due to the child’s language delay [2]. Indeed, children with ASD, often, struggle with more or less pronounced language deficits, particularly in language use during communicative interactions [3]. In parent–child interactions, the form, content, and use of language are fundamental components. The form and content of language (e.g., phonology, morphology, syntax, and semantics) represent structural language, and the appropriate use of language in social contexts represents the level of pragmatic skills, including turn-taking skills and initiating and maintaining a conversation [4,5]. Structural language and pragmatic skills, together, constitute interactive language.

Previous studies have shown that children with ASD have interactive language deficits that involve not only structural language but also pragmatic skills [6,7,8,9,10]. Regarding structural language, many children with ASD have difficulty with vocabulary comprehension and expression, and they lack lexical diversity, produce shorter utterances, and use fewer grammatically complex sentences [11,12]. In terms of pragmatic skills, children with ASD are less likely to participate in interactions and have a weaker ability to initiate and maintain conversations [13,14]. Previous studies have, typically, examined structural language and pragmatic skills separately. They found that structural language deficits are common, whereas pragmatic impairments are a recognized feature of ASD, regardless of language level or age [15]. However, previous work has rarely been concerned with the relationships and interactions between structural language deficits and pragmatic skill deficits, in children with ASD.

Meanwhile, an important external factor in children’s language development is the language input from caregivers (e.g., parents). In studies of typically developing (TD) children, both the quantity (e.g., number of words) and quality (e.g., type of words) of language input can be used to predict children’s language development [16]. A similar relationship has been found in children with ASD, that is, the caregivers’ language input has an important influence on the language development in children with ASD [17,18,19]. For example, language development in children with ASD is closely related to the number of words heard in the first year of life [19], the grammatical complexity of language input in early childhood [20], and the positive response and expansion of utterances by caregivers throughout childhood [17]. While several studies have compared language input between children with ASD and TD children, finding that parents’ pragmatic skills may differ from structural language [21,22,23], few studies have focused on input differences in pragmatic skills, between children with ASD and TD children.

Standardized measures, such as the Peabody Picture Vocabulary Test (PPVT-IV) [24], the Comprehensive Assessment of Spoken Language [25], and the Test for Reception of Grammar (TRG) [26], have been, widely, used to measure how children with ASD perform using vocabulary and simple/complex sentences. Although children with ASD performed well on standardized measures and did not differ significantly from age-matched TD children [10,27], standardized measures have been shown to have limitations. For example, Barokova and Tager-Flusberg [28] found that standardized measures may misrepresent the oral language abilities of children with ASD, since the social and communicative context may, directly, influence language performance. Thus, there is an urgent need to develop other measures, such as natural language samples (NLS), which assess children’s language use in natural contexts that carry detailed language information about structural language and pragmatic skills, which can provide a more comprehensive insight into language use than standardized measures.

Natural language samples, usually recorded in spontaneous conversations between children and parents, represent the language that children use in daily life. These natural language samples are, usually, produced in different contexts that may have different effects on children’s language performance. For example, compared with the daily communication context, the playing context induces more interactive utterances of children with ASD, which lead to more turn-takings [29]. The reading context induces more diversified vocabulary as well as longer and more complex sentences than both the daily communication and playing context [21,30]. However, current research is, mainly, focused on the playing and reading contexts. More contexts are needed to fully understand the interactive language performance of children with ASD.

Therefore, the overarching goal of this study was to investigate the interactive language of children with ASD in different contexts. To this end, we used a semi-structured format to survey interactive language in four contexts, playing, drawing, reading, and free talking, and analyzed (a) the structural language, by evaluating vocabulary and utterance, and (b) the pragmatic skills by assessing turn-taking. Specifically, we attempted to address the following issues:

(1) How do structural language and pragmatic skills used in communicative interaction differ between children with ASD and TD children?
(2) How do different contexts affect the interactive language of children with ASD?
(3) How does maternal input affect the language output of children with ASD?

## 2. Method

### 2.1. Participants

In total, 28 Mandarin-speaking children with ASD and 25 TD children were recruited and tested. Three participants from the ASD group were excluded because they had not completed the tasks. There were 3 girls and 22 boys in both the ASD and TD groups. The parents of the participants signed an informed consent form, approved by Nanjing Normal University. The 25 ASD children were from Nanjing Mingxin Children Wisdom Education and Training Centre (Nanjing, China), while the 25 TD children were from Nanjing Guanyun International Kindergarten (Nanjing, China). The children with ASD had been previously diagnosed by Nanjing Brain Hospital and were able to communicate verbally with their mothers and complete language tasks.

We assessed receptive and expressive vocabulary to ensure that the two groups had similar vocabulary skills using the Diagnostic Receptive and Expressive Assessment of Mandarin (DREAM) [31]. The DREAM was a standardized oral language test for children ages 2.5 to 7.9. It is similar to the Peabody Picture Vocabulary Test (PPVT), which was often used as an index of a child’s verbal mental age. The test consisted of receptive vocabulary and expressive vocabulary. The receptive vocabulary was tested by a picture-identifying task, while the expressive vocabulary was tested by a picture-naming task. The Cronbach’s alpha of DREAM was 0.85 [31]. The children’s nonverbal IQ was measured using the Primary Test of Nonverbal Intelligence (PTONI) [32]. We, also, recorded the parents’ educational level with a questionnaire, completed by the mothers of the ASD and TD groups. Educational level was ranked on a five-point Likert scale, from 1 (elementary school) to 5 (master’s or doctoral degree).

Table 1 provides basic information about the ASD and TD groups. The ASD and TD groups differ, significantly, in age (t = 10.198, *p* < 0.001), while there are no significant differences in receptive vocabulary, expressive vocabulary, parents’ educational level, and nonverbal IQ (*p_s_* > 0.05).

### 2.2. Procedure

Mother–child interactions, of both children with ASD and TD children, were studied in a private room in their kindergarten. Four contexts were conducted during the interactions, including playing, drawing, reading, and free talking. These four contexts represent the typical activities of children’s daily life. In many studies, these four contexts are also frequently used to elicit spontaneous conversation [21,33]. The details of the four contexts are provided below:

*Playing context*. We provided a picture of a slide and some plastic blocks. Then, mother and child were asked to build a similar slide together, based on the picture.

*Drawing context*. We provided crayons and drawing paper. Then, mother and child were asked to draw a picture together (without content instructions).

*Reading context*. We provided a wordless picture book (e.g., *Frog, Where Are You?* [34]), for mother and child to read together and discuss the content.

*Free talking*. Mother and child talk freely, with nothing provided.

The mother–child interaction was filmed with a video camera. The researchers were not allowed to intervene in the interaction. Then, the content of the first 5 min of each context was selected as a valid corpus, totaling 20 min for four contexts. The corpus for the 25 children in each group was 500 min long.

### 2.3. Transcription and Measurement

Two transcribers used the Codes for Human Analysis of Transcripts (CHAT), developed by MacWhinney [35], to transcribe the spontaneous conversation of mother and child into texts (approximately 110,000 Chinese characters). The transcriptions included all subordinate clauses or non-clausal structures attached to or embedded in them, as well as all functional units that did not have sentence-like status (e.g., ‘Yeah, OK’). The reliability of the transcriptions was assessed, by calculating the agreement between the two transcribers using a random selection of 10% of the total recorded material, and the cross-transcriber consistency was up to 95%.

Subsequently, these transcriptions were analyzed using Computerized Language Analysis (CLAN) [35] from two aspects: structural language and pragmatic skills. Structural language included vocabulary level and utterance level. The measures on the vocabulary level included the total number of words (TNW), the number of different words (NDW), the mean length of utterance (MLU), and the vocabulary diversity (vocD). TNW referred to the total number of words produced by each participant. NDW referred to the total number of unduplicated words produced by each participant (e.g., “chi1” [eat] may occur three times in the sample but only counts as one type). vocD was the ratio of different words used by a speaker and was considered an indicator of vocabulary diversity [36]. TNW, NDW, and vocD were generated using the program CLAN. The utterance level included the total number of utterances (TNU), the mean length of utterance (MLU), and the mean length of the longest five utterances (MLU-5). MLU was a measure of children’s sentence complexity and was calculated by dividing the total number of words by the number of utterances in each speech. Here, MLU-5 was calculated only for the longest five utterances. The pragmatic skills referred to the turn level. The measures on the turn level included the total number of turns (TNT), the utterances per turn (UPT), and the words per turn (WPT). TNT, UPT, and WPT were also calculated, using the CLAN program.

### 2.4. Analysis

Since all variables were not normally distributed, Mann–Whitney U tests were performed to compare the two groups. Moreover, mixed ANOVAs were used to examine the main and interaction effects of the group as well as the context of the measures of interactive language. Spearman’s rank correlation analyses were conducted, to examine the relationships between maternal input and children’s language output. Correlation analysis was performed using R statistical software (v. 3.6.1) (https://www.r-project.org accessed on 20 January 2022).

## 3. Results

### 3.1. Comparisons of ASD and TD Groups in Structural Language and Pragmatic Skills Used in Communicative Interaction

The results for structural language and pragmatic skills, used in communicative interaction, can be found in Table 2. Regarding vocabulary level, there were no significant differences between the ASD and TD groups in the total number of words (TNW), the number of different words (NDW), and the vocabulary diversity (vocD). However, in the utterance level, the ASD group was significantly lower than the TD group in the mean length of utterance (MLU) and the mean length of the longest five utterances (MLU-5). These results suggest that, although children with ASD and TD children had similar vocabulary, the grammatical skills of TD children were higher than those of children with ASD. On the turn level, we found significant differences in the total number of turns (TNT), the number of utterances per turn (UPT), and the number of words per turn (WPT), suggesting that children with ASD exhibited fewer and linguistically fewer complex turns.

### 3.2. Main and Interaction Effects of Group and Context on Interactive Language

For the effects of group (ASD/TD) and context (playing/drawing/reading/free talking) on the interactive language measures (vocabulary/utterance/turn), Table 3 shows the descriptive statistics as well as the results of the mixed ANOVAs.

*Vocabulary*. We found that (1) the main effect of context (C) was significant in total number of words (TNW), number of different words (NDW), and vocabulary diversity (vocD). However, the main effect of group (G) was significant only in vocD, and the two-way interaction effects of group and context (G × C) were significant only in NDW. (2) For the G×C interaction, we were, primarily, concerned with how the context effect affected the vocabulary output of the two groups. In the ASD group, the NDW was significantly greater in the reading context than in the drawing and free talking contexts. Similarly, vocD was significantly greater in the reading context than in the playing, drawing, and free talking contexts. In the TD group, both NDW and vocD had significantly higher values in the reading context than in the drawing and free talking contexts. These results suggest that children, regardless of being in the ASD or TD group, used more diverse lexical resources in reading than in other activities.

*Utterance*. Table 3 shows that (1) the main effect of context (C) was significant in the total number of utterances (TNU), mean length of utterance (MLU), and mean length of the longest five utterances (MLU-5). However, the main effect of group (G) was significant only in MLU. We, also, found that the two-way interaction effects of group and context (G × C) were significant in TNU and MLU. (2) For context differences, we found that in both the ASD and TD groups, TNU was significantly lower in the playing, drawing, and reading contexts than in the free talking context, while MLU was significantly larger in the playing, drawing, and reading contexts than in the free talking context. These results suggest that daily activities such as playing, drawing, and reading promote children’s language output with longer (see MLU) utterances.

*Turn*. We found that (1) the main effects of group (G), context (C), and the two-way interaction effects of group and context (G × C) on turn were all significant in utterances per turn (UPT) and words per turn (WPT). However, the main effect of context (C) was significant only in the total number of turns (TNT). (2) For context differences, we found that TNT was larger in the free talking context than in the context of playing, drawing, and free talking, regardless of group differences. However, WPT was lower in the free talking context than in the playing, drawing, and reading contexts. These results suggest that daily talking is an important way to improve children’s ability to take turns (see TNT). However, this does not mean that more utterances or words were used in each turn in the free talking context than in other contexts (see UPT and WPT).

### 3.3. Correlation Analysis

The relationship between the maternal language input and the interactive language output of children with ASD is shown in Table 4. In terms of structural language (TNW, NDW, ..., MLU-5), we found that the vocabulary diversity (vocD) of children with ASD was significantly correlated with mothers’ vocD (*r*_s_ = 0.71, *p* < 0.01), and children’s total number of utterances (TNU) was significantly correlated with mothers’ number of different words (NDW, *r*_s_ = 0.41, *p* < 0.05). However, no significant correlations were found between the language of children with ASD and the language of their mothers in the total number of words (TNW), number of different words (NDW), mean length of utterance (MLU), and mean length of longest five utterances (MLU-5), suggesting that the structural language of children with ASD was less likely to be influenced by the structural language input from their mothers. As for pragmatic skills, the total number of turns (TNT) of children with ASD were significantly correlated with mothers’ TNT, NDW, and TNW, suggesting that the pragmatic skills of children with ASD were influenced not only by their mothers’ structural language but also by their mothers’ pragmatic skills.

## 4. Discussion

### 4.1. Structural Language and Pragmatic Skill Deficits in Children with ASD

The aim of this study was to investigate the structural language and pragmatic skills of children with ASD in different contexts. We found that compared with TD children, children with ASD and a similar vocabulary level were characterized by impairments in pragmatic skills rather than in structural language. For example, we found that children with ASD did not show impairments in the quantity (e.g., number of words (TNW)) or the quality of words (e.g., number of different words (NDW) and vocabulary diversity (vocD)) during mother–child interaction. This result differs from previous studies, such as Anderson et al. [37] and Woynaroski [38], who claimed that children with ASD had severe impairments in vocabulary quantity and quality. One possible explanation for this could be the use of different measurement methods. For example, previous studies, often, used standardized measures (e.g., PPVT), so children with ASD might have difficulty completing the tasks because of social impairments or attention deficits. In contrast, the language samples we used were obtained from natural conversations, so children with ASD were likely to speak more words and have more diverse vocabularies. There are, however, other possible explanations. For example, even if children have been diagnosed with ASD, the severity may vary widely. According to the vocabulary level and nonverbal IQ of children with ASD in Table 1, the children with ASD we selected belong to the type with verbal fluency and intact general nonverbal abilities, whereas, children with ASD in other studies may belong to the types with minimal verbal or speech phrase [37,38], which could explain why there are no deficits in vocabulary use of children with ASD in our study.

Regarding pragmatic skills, the total number of turns (TNT), the number of utterances per turn (UPT), and the number of words per turn (WPT) in children with ASD are significantly lower than in TD children (Table 2). Previous studies have also found that children with ASD used fewer turns and had more difficulty maintaining topics than TD children [39,40], which could be used as an important indicator for diagnosing autistic. One possible reason for this is that children with ASD have difficulty with perspective taking [41], circumscribed interests [42], and the ability to predict what the listener already knows or wants to know [43]. As a result, children with ASD have difficulty managing topics and are less likely to take turns during interactions.

### 4.2. The Effects of Context on Interactive Language of Children with ASD

Our study found that context influenced both structural language and pragmatic skills in children with ASD. For example, we found that children with ASD exhibited higher number of different words (NDW) and vocabulary diversity (vocD) in the reading context than in the other contexts. This result is consistent with Demir-Vegter [21], who found that the reading context played an important role in the development of lexical richness in children with ASD. In fact, mothers are more likely to provide written language and complicated vocabulary in the reading context [44]. As a result, children are more likely to learn new words, making their vocabulary more diverse, than in other contexts.

We also found that the mean length of utterance (MLU) of children with ASD was higher in the playing, drawing, and reading contexts than in the free talking context, suggesting that children with ASD used more grammatically complex sentences in such contexts. This result may be explained by the fact that children are in the developmental stage of concrete thinking [45]. Given a particular object or picture, children may have a lower cognitive load and are more likely to produce high-quality language samples. Another possible explanation could be related to joint attention, which refers to a conscious attention of both participants to the same object or event [46]. In our case, mother–child interactions in the contexts of playing and drawing use a specific medium and might be characterized by a higher level of joint attention, so that more cognitive resources can be released for verbal interaction, leading to a higher level of linguistic complexity.

In the free talking context, we found that children with ASD had a higher number of turns but a lower number of utterances per turn (UPT) than in other contexts. Although an increase in the number of turns, inevitably, leads to a decrease in the length of each turn in the same period of time, there might be other explanations, for example, mothers in the playing, drawing, and reading contexts tended to use declarative or explanatory utterances, to which children often do not need to respond, whereas mothers in the free talking context often used interrogative utterances, to which children need to give a clear response, resulting in a higher number of turns.

### 4.3. Correlation between Maternal Input and Interactive Language of Children with ASD

Perhaps the most compelling finding is that the correlations between structural language and maternal input in children with ASD were not as high as revealed in other studies, such as Cristofaro and Tamis-LeMonda [47] and Fusaroli et al. [22]. For example, Fusaroli et al. [22] found that maternal structural language (e.g., MLU, a measure of grammatical complexity) can be used to predict language development in children with ASD. One possible explanation could be that the children with ASD in our study are older (>6 years old) than those examined in other studies. When children are young (0~6 years old), language acquisition occurs, mainly, through their mother [6,48], leading to a strong relationship between maternal input and children’s language development. However, as children get older (>6 years old, as in this study), the children’s structural language has developed to a certain extent, so the mothers’ language input is no longer critical for the children’s language acquisition.

Meanwhile, a, relatively, obvious relationship was found between pragmatic skills and maternal input. This is because children with ASD have obvious deficits in their pragmatic skills and rely on their mothers to constantly initiate and maintain conversations during interactions. Therefore, we hypothesize that, although the structural language of children with ASD may improve with age, their pragmatic skills deficits are more pronounced than those of TD children.

### 4.4. Limitations

There are several limitations of the current study. First, this study sampled interactive language between mother and child in kindergarten, whereas home may be a better ecological site. Therefore, future studies should consider collecting interactive language samples, from children with ASD, at home. Second, group comparison (TD children and children with ASD) and sample representativeness can be improved. For example, we selected the TD children and the children with ASD who had a similar vocabulary level, but factors such as age were not fully taken into account. Finally, the language development of children with ASD may be influenced by the quality of parent-child interactions, such as parents’ use of supportive language techniques (e.g., expansions, rephrasing, open-ended questions). Therefore, in future work, we should fully consider the influence of the quality of parent–child interactions on children’s pragmatic skills.

## 5. Conclusions

We aimed to investigate the interactive language of children with ASD in different contexts. We found that, although deficits in structural language (e.g., vocabulary and utterance) were not pronounced compared to TD children, children with ASD, clearly, had deficits in pragmatic skills. We also found that contexts play a key role in the use of interactive language by children with ASD. Specifically, we found that (a) the reading context is essential for the development of a diverse vocabulary, (b) the playing and drawing contexts makes an important contribution to the formation of complex sentences, and (c) the free talking context helps to generate more turns. Perhaps the most interesting aspect of this study is that the correlations between structural language and maternal input in children with ASD were not as high as revealed in previous studies, while a, relatively, obvious relationship was found between pragmatic skills and maternal input. Therefore, we propose that: (1) pragmatic skills should be considered, in the assessment of children with suspected ASD, (2) the influence of context on pragmatics should be taken into account, when assessing the pragmatic development of children with ASD, and (3) more attention should be paid to improving the mother’s pragmatic skills, in the intervention of children with ASD.

Our research also has important implications for language intervention in children with ASD. For example, parents should teach high-quality pragmatic skills for children with ASD, in order to better support their language development. Parents should also pay attention to their children’s communication intentions and increase turn-taking awareness, to improve the feedback skills of children with ASD. In addition, different contexts could be used to promote the language development of children with ASD. For example, parents should, intentionally, increase language interaction during daily activities, or have more conversations about recalling one day of life in kindergarten. Future research could include a long-term follow-up study of mother–child interactions, to examine the effects of the quality of maternal interaction on children’s pragmatic skills and compare the differences between the pragmatic skills of the mothers of children with ASD and the mothers of TD children.

## Figures and Tables

**Table 1:** Characteristics of ASD (*n* = 25) and TD (*n* = 25) groups.

	Months	Receptive Vocabulary	Expressive Vocabulary	Parents Education	Nonverbal IQ
ASD	67.84 (10.77)	42.92 (4.99)	31.88 (7.93)	3.72 (0.67)	118.6 (10.82)
TD	44.52 (3.85)	42.80 (5.39)	32.76 (6.82)	3.84 (0.59)	100.4 (10.91)
t	10.198 ***	0.082	0.421	0.318	0.098

Note: *** *p* < 0.001, ASD = autism spectrum disorder, TD = typically developing.

**Table 2:** Effects of the group (ASD/TD) on structural language and pragmatic skills in communicative interaction: descriptive statistics and results of Mann–Whitney U tests.

		ASD (*n* = 25)	TD (*n* = 25)	U	*p*
		Mean (SD)	Mean (SD)		
Vocabulary	TNW	366.80 (143.43)	415.56 (116.98)	238.50	0.151
	NDW	162.88 (43.46)	176.40 (33.61)	258.50	0.295
	vocD	77.01 (18.34)	84.55 (18.25)	249.00	0.218
Utterance	TNU	153.88 (43.79)	159.36 (29.02)	282.00	0.674
	MLU	2.44 (0.53)	2.87 (0.51)	164.50	<0.01
	MLU-5	6.93 (1.86)	7.88 (1.46)	196.00	<0.05
Turn	TNT	143.84 (40.16)	155.48 (27.16)	291.50	<0.05
	UPT	1.03 (0.10)	1.09 (0.07)	161.50	<0.01
	WPT	2.45 (0.77)	2.89 (0.75)	205.00	<0.05

Note: ASD = autism spectrum disorder, TD = typically developing, TNW = total number of words, NDW = number of different words, vocD = vocabulary diversity, TNU = total number of utterances, MLU = mean length of utterance, MLU-5 = mean length of the longest five utterances, TNT = total number of turns, UPT = utterances per turn, WPT = words per turn.

**Table 3:** Effects of group (ASD/TD) and context (playing/drawing/reading/free talking) on interactive language measures (vocabulary/utterance/turn): Descriptive statistics and results of mixed ANOVAs.

	Measures	Group Mean (SD)								Mixed ANOVAs			Post-Hoc Test	
		ASD (*n* = 25)				TD (*n* = 25)				G	C	G × C	ASD	TD
		P	D	R	FT	P	D	R	FT	*F* (*η^2^*)	*F* (*η^2^*)	*F* (*η^2^*)		
Vocabulary	TNW	105.48 (9.82)	84.28 (10.19)	103.60 (8.84)	79.08 (6.79)	96.92 (9.35)	111.12 (7.70)	112.4 (9.87)	84.52 (3.17)	1.39 (0.03)	7.63 *** (0.14)	3.29 (0.07)	P, R > D, FT	D, R > P, FT
	NDW	53.04 (4.16)	41.88 (3.47)	57.71 (4.39)	44.80 (3.04)	55.32 (3.77)	54.68 (3.39)	65.80 (3.69)	47.80 (3.78)	3.74 (0.07)	7.68 *** (0.14)	1.36 * (0.26)	P, R > D, FT	R > P, D, FT
	vocD	38.08 (3.36)	31.31 (2.80)	50.67 (5.86)	39.16 (4.32)	47.94 (4.58)	38.83 (3.11)	54.83 (5.40)	41.18 (3.23)	4.09 * (0.08)	9.17 *** (0.16)	0.19 (0.004)	R > P, D, FT	R > P, D, FT
Utterance	TNU	41.92 (12.57)	32.44 (11.65)	40.00 (12.10)	47.60 (11.09)	33.36 (8.31)	38.48 (10.01)	39.2 (14.17)	46.68 (9.78)	2.39 (0.05)	517.16 *** (0.92)	5.15 * (0.10)	FT > P, D, R	FT > P, D, R
	MLU	2.42 (0.71)	2.46 (0.81)	2.67 (1.05)	1.70 (0.80)	2.85 (0.75)	2.85 (0.87)	3.12 (1.42)	1.85 (0.99)	5.16 *** (0.10)	452.15 * (0.90)	3.35 * (0.07)	P, D, R > FT	P, D, R > FT
	MLU-5	5.54 (1.73)	4.68 (1.75)	4.23 (0.94)	5.19 (1.69)	6.02 (1.48)	5.96 (1.47)	5.05 (1.75)	6.04 (1.68)	0.17 (0.004)	358.26 *** (0.88)	0.04 (0.001)	P, FT> D, R	P, D, FT> R
Turn	TNT	35.68 (12.86)	34.68 (14.33)	37.44 (12.53)	43.08 (13.05)	29.20 (7.56)	34.84 (9.58)	36.84 (11.94)	44.72 (8.87)	0.30 (0.24)	15.19 *** (0.24)	1.97 (0.04)	FT > P, D, R	FT> P, R, D,
	UPT	1.27 (0.50)	1.25 (1.62)	1.10 (0.25)	1.16 (0.26)	1.15 (0.10)	1.11 (0.13)	1.09 (0.17)	1.04 (0.05)	70.15 *** (0.59)	49.53 *** (0.51)	63.15 *** (0.57)	n.s.	n.s.
	WPT	2.95 (1.01)	3.18 (2.25)	2.87 (1.05)	2.06 (1.39)	3.30 (1.02)	3.25 (1.35)	3.36 (1.47)	1.95 (1.15)	185.56 *** (0.69)	108.50 *** (0.69)	111.74 *** (0.70)	P, D, R > FT	P, D, R > FT

Note: * *p* < 0.05, *** *p* < 0.001, n.s. = non-significant. ASD = autism spectrum disorder, TD = typically developing, P = playing, D = Drawing, R = reading, FT = free talking, G = Group, C = Context.

**Table 4:** Spearman’s rank correlations between the interactive language of mothers (-M) and children with ASD (-C).

	TNW-C	NDW-C	vocD-C	TNU-C	MLU-C	MLU5-C	TNT-C	UPT-C	WPT-C
TNW-M	0.34	0.23	0.31	0.37	−0.06	0.22	0.44 *	−0.25	−0.10
NDW-M	0.20	0.20	0.08	0.41 *	−0.17	0.03	0.46 *	−0.18	−0.27
vocD-M	−0.18	−0.39	0.71 **	−0.26	−0.37	−0.13	−0.18	−0.36	−0.40 **
TNU-M	0.16	0.27	−0.02	0.23	0.24	0.09	0.15	0.21	0.18
MLU-M	−0.26	−0.29	−0.07	−0.37	−0.18	−0.16	−0.39	0.05	−0.12
MLU5-M	−0.18	−0.29	0.09	−0.39	−0.08	−0.05	−0.42 *	0.07	−0.05
TNT-M	0.10	0.11	0.04	0.38	−0.28	−0.12	0.47 *	−0.33	−0.41 *
UPT-M	0.10	−0.01	0.33	−0.04	0.00	0.27	−0.01	0.02	0.03
WPT-M	0.20	0.07	0.35	0.25	0.00	0.33	0.33	−0.28	−0.12

Note: * *p* < 0.05, ** *p* < 0.01.

## Data Availability

Data is contained within the article.
